# ∼14 000 years of geochemical and isotopic data from Lake Simcoe, Canada

**DOI:** 10.1016/j.dib.2022.108541

**Published:** 2022-08-14

**Authors:** R.M. Doyle, N. Bumstead, C.F.M. Lewis, F.J. Longstaffe

**Affiliations:** a Department of Earth Sciences, Biological and Geological Sciences, The University of Western Ontario, 1151 Richmond St N, London, ON N6A 5B7, Canada; b AMGC Research Group, Vrije Universiteit Brussel, Pleinlaan 2, Brussels 1050, Belgium; c Geological Survey of Atlantic Canada, Bedford Institute of Oceanography, Box 1006, 1 Challenger Drive, Dartmouth, Nova Scotia, B2Y 4A2, Canada

**Keywords:** Ostracods, Laurentian Great Lakes region, Glacial meltwater, 8.2ka event, Holocene, Pleistocene-Holocene transition, Lake Algonquin

## Abstract

This dataset contains measurements of modern water and ancient core materials from Lake Simcoe, the fourth largest lake wholly in Ontario, Canada. These data consist of: *(i)* oxygen, hydrogen and carbon isotope (*δ*^18^O, *δ*^2^H and *δ*^13^C) compositions for modern water samples; *(ii)* physical measurements of one piston core, PC-5; *(iii) δ*^13^C and *δ*^18^O values of ostracods collected from PC-5, and *(iv) δ*^13^C and *δ*^18^O values of ancient DIC and water, respectively, inferred from item *(iii)*. Physical measurements performed on core PC-5 include magnetic susceptibility, mineralogy and grain size. Mass accumulation rates are also reported. These data will be of interest to those aiming to better characterize the timing and pathway of meltwater flow during and following deglaciation of the Laurentide Ice Sheet in the Laurentian Great Lakes region. These data will also be useful to researchers investigating the influence of deglaciation on the oxygen and carbon isotope systematics of ancient lake environments. A discussion of these data is available in “A ∼14 000-year record of environmental change from Lake Simcoe, Canada” [1].

## Specifications Table

**Table:** 

Subject	Geochemistry and Petrology, Environmental Science
Specific subject area	Isotopic and geochemical data from lake sediments, including ostracods.
Type of data	.xlsx and .docx files
How the data were acquired	Modern water samples from Lake Simcoe were analyzed using *(i)* a PicarroⓇ L2120-i *δ*^2^H and *δ*^18^O Analyser and *(ii)* a Thermo Scientific™ GasBenchⓇ II connected to a Thermo Scientific™ Delta^plus^ XL™ continuous flow isotope ratio mass spectrometer (IRMS) and a heater block equipped with a CombiPalⓇ autosampler. Ostracods collected from sediment core PC-5 were analyzed using *(i)* a Micromass MultiPrepⓇ device coupled to a VG OptimaⓇ dual-inlet isotope ratio mass spectrometer (IRMS) or *(ii)* a Thermo Scientific™ GasBenchⓇ II interfaced with a Thermo Scientific™ Delta^plus^ XL™ continuous flow IRMS. Sediment core PC-5 was analyzed using the following instruments: *(i)* a Malvern MastersizerⓇ 2000 laser grain-size analyzer, *(ii)* a GEOTEKⓇ multi-sensor core logger (MSCL) and *(iii)* a Rigaku High Brilliance Rotating Anode X-ray Diffractometer.
Data format	Raw.
Description of data collection	All samples were collected from Lake Simcoe. The water samples were collected at various depths in the lake from May 2009 to November 2011. All other data originate from sediment core PC-5 collected from the deepest, flattest part of the lake in June 2007.
Data source location	Lake Simcoe, Ontario, Canada, 44.4873, −79.4169
Data accessibility	[2] R.M. Doyle, N. Bumstead, C.F.M. Lewis, F.J. Longstaffe. Geochemical and isotopic analyses of a ∼14 000 year old sediment core collected from Lake Simcoe, Canada. Zenodo (2022) https://doi.org/10.5281/zenodo.6959403.
Related research article	[1] R.M. Doyle, N. Bumstead, C.F.M. Lewis, F.J. Longstaffe, A ∼14 000-year record of environmental change from Lake Simcoe, Canada, Quat. Sci. Rev. 292 (2022) 107667, doi:https://doi.org/10.1016/j.quascirev.2022.107667.

## Value of the Data

• These data may be compared with other proxy archives in the Laurentide Great Lakes region of North America to better characterize the timing and pathway of meltwater flow following deglaciation of the Laurentide Ice Sheet.
• These data are useful to researchers investigating the influence of deglaciation on the oxygen and carbon isotope systematics of ancient lake environments.
• These data could also be used as a baseline for relative temperature change in southern Ontario.
• These data are beneficial to researchers interested in contextualizing the recent eutrophication of Lake Simcoe against a backdrop of natural variation.

## 1 Data Description

These data include the stable isotope ratios (*δ*^18^O, *δ*^2^H and *δ*^13^C) of modern water from Lake Simcoe, as well as the stable isotope ratios (*δ*^18^O and *δ*^13^C) of ostracods in core sediments from Lake Simcoe. Physical measurements of sediment core PC-5 (e.g., grain size, magnetic susceptibility, mineralogy) are also provided. For plots of these data, refer to Doyle and colleagues [1]. This dataset is contained in one Microsoft Excel file:

LakeSimcoeData.xlsx – one data table containing all analytical measurements, including: *(i) δ*^18^O, *δ*^13^C and *δ*^2^H of modern waters; *(ii)* ostracod assemblages from core PC-5; *(ii) δ*^18^O and *δ*^13^C of ostracod valves in core PC-5; *(iii)* mineralogy of core PC-5; *(iv)* magnetic susceptibility of core PC-5; *(v)* grain size analysis of core PC-5. The *δ*^18^O and *δ*^13^C of ancient water and DIC, respectively, are inferred from isotopic analyses of ostracod valves and are also reported in this datasheet.

## 2 Experimental Design, Materials and Methods

Analysis of *δ*^2^H and *δ*^18^O of modern waters: Unfiltered water samples were removed from the refrigerator and left to sit until they warmed to room temperature. 1 mL of water was pipetted into a 2 mL glass vial for analysis. Analysis of *δ*^2^H and *δ*^18^O of modern waters using a PicarroⓇ L2120-i *δ*^2^H and *δ*^18^O Analyser – Calibration of *δ*^2^H and *δ*^18^O to VSMOW was achieved using LSIS standards Heaven (accepted *δ*^2^H: +88.7‰; accepted *δ*^18^O: ‒0.27‰) and LSD (accepted *δ*^2^H: ‒161.8‰; accepted *δ*^18^O: ‒22.57‰). Analytical accuracy and precision were evaluated using LSIS standards MID (accepted *δ*^2^H: ‒108.1‰; accepted *δ*^18^O: +13.08‰) and EDT (accepted *δ*^2^H: ‒56‰; accepted *δ*^18^O: ‒7.27‰). The *δ*^2^H results for MID and EDT were ‒108.00 ± 0.67‰ (*n* = 35) and ‒54.68 ± 1.17‰ (*n* = 111), respectively, which compare well with accepted values and expected reproducibility. The *δ*^18^O results for MID and EDT were ‒13.01 ± 0.14‰ (*n* = 35) and ‒7.22 ± 0.18‰ (*n* = 111), respectively, which compare well with accepted values and expected reproducibility. The accepted values for all LSIS standards were determined previously by direct calibration to a VSMOW- and SLAP-scale.

Analysis of *δ*^13^C_DIC_ of modern waters: For samples, five drops of 100% concentrated orthophosphoric acid were added to the bottom of the glass vials, which were then septum-sealed and flushed with He for 5 min. One (1) mL of sample was then injected into the flushed vial using a 1 mL syringe. The vials were reacted in the GasBenchⓇ heater block at 35 °C overnight prior to isotopic measurements. The produced gas was then transported automatically to the IRMS using an autosampler. For each standard, 0.25 mg was weighed into the bottom of a glass vial, and the vial was placed in a horizontal position. Concentrated orthophosphoric acid (100%) was then added to the top of the vial such that the acid was separated from the standard powder. A septum cap was then attached to the vial and tightened. Next, the vial was flushed with He for 5 min at room temperature. The vial was then turned upright, thus allowing the acid to react with the standard powder. The vial was then immediately placed in the GasBenchⓇ heater block overnight reacting at 35 °C. The evolved gas was then automatically transferred to the IRMS using an autosampler. Analysis of *δ*^13^C_DIC_ of modern waters was conducted using a Thermo ScientificTM GasBenchⓇ II coupled to a Thermo Scientific™ Deltaplus XL™ IRMS. Calibration of *δ*^13^C to VPDB was achieved using NBS-18, NBS-19 and Suprapur. Accuracy and precision of analyses were evaluated using the *δ*^13^C of WS-1. The *δ*^13^C results for WS-1 were 0.80 ± 0.11‰ (*n* = 5), which compare well with accepted values and expected reproducibility.

Analysis of *δ*^13^C and *δ*^18^O of ostracods: To collect ostracods, each interval of sediment was first wet-sieved. The sieved material and ostracods were separated under a binocular microscope using statically charged camel hairs. Ostracod valves were then identified and cleaned with bleach to remove organic material adhered to the valves. The *δ*^13^C and *δ*^18^O of most ostracods were measured using a Micromass MultiPrepⓇ coupled to a dual-inlet VG OptimaⓇ Isotope Ratio Mass Spectrometer (IRMS). Calibration of *δ*^13^C to VPDB was achieved using NBS-19 (accepted value: *δ^13C^* = + 1.95‰) and Suprapur (accepted value: ‒35.28‰). Accuracy and precision of analyses were evaluated using the *δ*^13^C of NBS-18 (accepted value: ‒5.0‰) and LSIS standard WS-1 (accepted value: +0.76‰). The *δ*^13^C results for NBS-18 and WS-1 were –5.01 ± 0.07‰ (*n* = 41) and +0.72 ± 0.08‰ (*n* = 14), respectively, which compare well with accepted values and expected reproducibility. Calibration of *δ*^18^O to VSMOW was achieved using NBS-19 (accepted value: +28.65‰) and NBS-18 (accepted value: +7.20‰). Analytical accuracy and precision were evaluated using Suprapur (accepted value: +13.25‰) and WS-1 (accepted value: +26.23‰). The *δ*^18^O results for Suprapur and WS-1 were +13.28 ± 0.17‰ (*n* = 35) and +26.20 ± 0.11‰ (*n* = 14), respectively, which compare well with accepted VSMOW-SLAP calibrated values and expected reproducibility. The *δ*^13^C and *δ*^18^O of all remaining ostracods were measured using a Thermo Scientific™ GasBenchⓇ II coupled to a Thermo Scientific™ Deltaplus X™ IRMS. Calibration of *δ*^13^C to VPDB was achieved using NBS-19 and Suprapur. Accuracy and precision of analyses were evaluated using the *δ*^13^C of NBS-18 and WS-1. The *δ*^13^C results for NBS-18 and WS-1 were –4.99 ± 0.12‰ (*n* = 8) and +0.91 ± 0.28‰ (*n* = 6), respectively, which compare well with accepted values and expected reproducibility. Calibration of *δ*^18^O to VSMOW was achieved using NBS-19 and NBS-18. Analytical accuracy and precision were evaluated using Suprapur and WS-1. The *δ*^18^O results for Suprapur and WS-1 were +13.30 ± 0.34‰ (*n* = 8) and +26.03 ± 0.71‰ (*n* = 6), respectively, which compare well with accepted values and expected reproducibility. Methods for converting the *δ*^13^C and *δ*^18^O to estimates of *δ*^13^C_DIC_ and *δ*^18^O_lake water_ are provided in the main research article [1].

Grain-size analysis of PC-5: Grain-size analysis of samples from each core was performed using a Malvern MastersizerⓇ 2000 laser grain-size analyzer hosted in the Control and Crystallization of Pharmaceuticals Laboratory (CCPL) at The University of Western Ontario.

In preparation for grain-size analysis, samples from PC-5 were disaggregated and treated with 15 mL of 0.3% bleach at 65 °C for at least 24 h to remove organic matter. The bleach was then removed by repeatedly rinsing each sample with distilled water. Finally, 10 mL of sodium hexametaphosphate solution, a dispersing agent, was added to each sample and samples were analyzed using the MastersizerⓇ.

Analysis of the magnetic susceptibility (MS) of PC-5: Prior to the analysis of MS, sediment core PC-5 was rinsed with distilled water and gently scraped horizontally using a spatula. MS was assessed using a GEOTEKⓇ multi-sensor core logger (MSCL) in the Lake and Reservoir Systems Research Facility (LARS) at The University of Western Ontario.

Analysis of sediment mineralogy of PC-5: Samples from PC-5 were freeze-dried and homogenized using a rubber mortar and pestle. The sample was then mounted onto an Al-backpack holder or a glass front-pack holder and analyzed using a Rigaku, high brilliance, rotating-anode X-ray diffractometer equipped with a graphite monochromator and CoKα radiation produced at 45 kV and 160 mA. Samples were scanned from 2° to 82° 2θ at a scanning rate of 10° 2θ/min. The abundance of each mineral was estimated using the background-subtracted peak height of its most intense diffraction. Crystallinity differences were account for using a form factor of x1, except for the (001) diffractions of kaolinite (x2), chlorite (x2) and illite (x4).

## Ethics Statement

This work did not involve human subjects, animal experiments, or data collected from social media platforms. The manuscript adheres to Elsevier's ethics in publishing standards.

## CRediT authorship contribution statement

**R.M. Doyle:** Conceptualization, Writing – original draft, Writing – review & editing, Methodology, Formal analysis, Visualization. **N. Bumstead:** Conceptualization, Investigation, Methodology, Writing – review & editing. **C.F.M. Lewis:** Writing – review & editing. **F.J. Longstaffe:** Supervision, Conceptualization, Writing – review & editing, Resources, Project administration, Funding acquisition.

## Declaration of Competing Interest

The authors declare that they have no known competing financial interests or personal relationships that could have appeared to influence the work reported in this paper.

## Data Availability

Geochemical and isotopic analyses of a ∼14 000 year old sediment core collected from Lake Simcoe, Canada (Original data) (Zenodo).

## Appendix Supplementary materials

Image, application 1
